# Bioengineering of spider silks for the production of biomedical materials

**DOI:** 10.3389/fbioe.2022.958486

**Published:** 2022-08-09

**Authors:** Daniela Matias de C. Bittencourt, Paula Oliveira, Valquíria Alice Michalczechen-Lacerda, Grácia Maria Soares Rosinha, Justin A. Jones, Elibio L. Rech

**Affiliations:** ^1^ Embrapa Genetic Resources and Biotechnology, National Institute of Science and Technology—Synthetic Biology, Brasília, DF, Brazil; ^2^ Department of Biology, Utah State University, Logan, UT, United States

**Keywords:** spider silk, spidroins, biomaterial, bioengineering, biomedical applications, synthetic biology

## Abstract

Spider silks are well known for their extraordinary mechanical properties. This characteristic is a result of the interplay of composition, structure and self-assembly of spider silk proteins (spidroins). Advances in synthetic biology have enabled the design and production of spidroins with the aim of biomimicking the structure-property-function relationships of spider silks. Although in nature only fibers are formed from spidroins, *in vitro*, scientists can explore non-natural morphologies including nanofibrils, particles, capsules, hydrogels, films or foams. The versatility of spidroins, along with their biocompatible and biodegradable nature, also placed them as leading-edge biological macromolecules for improved drug delivery and various biomedical applications. Accordingly, in this review, we highlight the relationship between the molecular structure of spider silk and its mechanical properties and aims to provide a critical summary of recent progress in research employing recombinantly produced bioengineered spidroins for the production of innovative bio-derived structural materials.

## Introduction

One of the major goals of material science is to create innovative bio-derived structural materials with tailor made properties. For this purpose, synthetic biology tools have been applied to explore the function and structure of different biological materials, such as proteins, polysaccharides, and polyesters (Bryksin et al., 2014; Keating and Young, 2019). Because of their biocompatibility, biodegradation and non-immunogenicity, bio-derived materials have numerous applications in the medical field and are playing a central role in biomedical engineering (Long et al., 2021; Lynch et al., 2021).

In this context, spider silks have emerged as potential candidates for many biomedical and biotechnological applications, due to their intrinsic ability to perform very specific biochemical, mechanical and structural functions (Vollrath, 2000; Vollrath and Porter, 2009; Spiess et al., 2010; Kiseleva et al., 2020). The understanding of the modular sequence architecture of spider silks proteins (spidroins) and its relationship with the mechanical properties of silks shows a clear explanation how nature overcomes the challenges in the production of high performance materials. Moreover, it opens the possibility to produce recombinant spider silk proteins (rSSP) inspired by their natural counterparts, but with specific properties for the development of innovative bio-derived structural materials (Römer and Scheibel, 2008; Saric and Scheibel, 2019).

**FIGURE 1:**
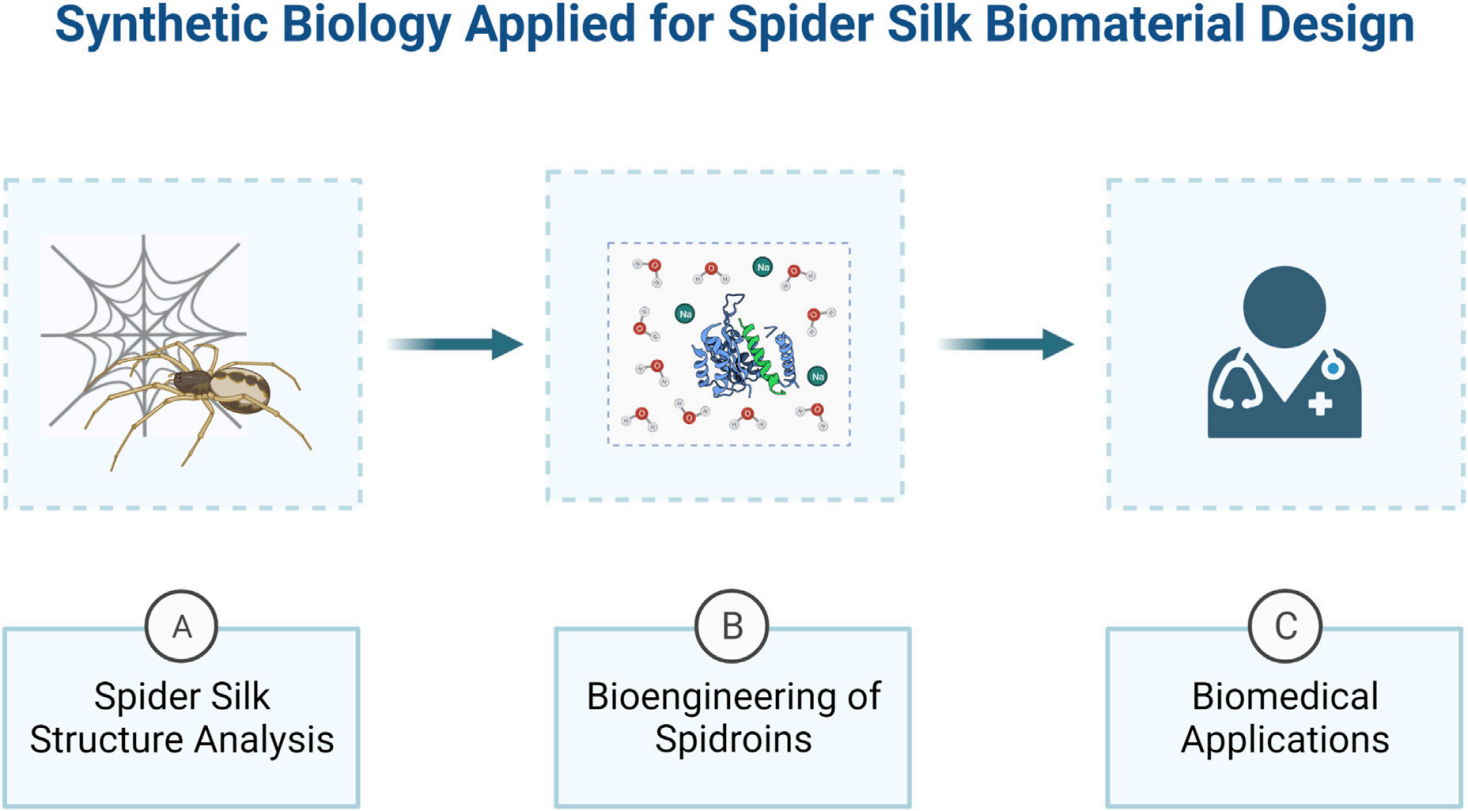
An overview of how synthetic biology can be applied for the development of spider silk bio-derived structural materials. Synthetic biology approaches are ideal to study spider silk proteins structure and mechanical properties **(A)** and engineering them to produce biomaterials with desirable properties **(B)**, in order to develop innovative biomedical devices **(C)**. Created with BioRender.com.

Several studies have already been developed aiming to directionally biodesign rSSP for different applications (Teulé et al., 2009: Spiess et al., 2010; Kucharczyk et al., 2018; Blamires et al., 2020; Bakhshandeh et al., 2021; Mulinti et al., 2021). In this regard, the interdisciplinary approach of synthetic biology has shown to possess the perfect set of tools to manipulate the biomaterial in all scales, since protein structure analysis until ultra-performance material design (Poddar et al., 2020) (Figure 1). With this in mind, here we present an overview about the relationship between spider silks protein structure and their mechanical properties, and how synthetic biology have been using this knowledge in the last years to develop innovative synthetic spider silk based biomaterials. Moreover, we also review the biomedical application of rSSP, along with the advances in the development of improved biological systems for the production of rSSP.

## Spider silks structure and functions

**FIGURE 2:**
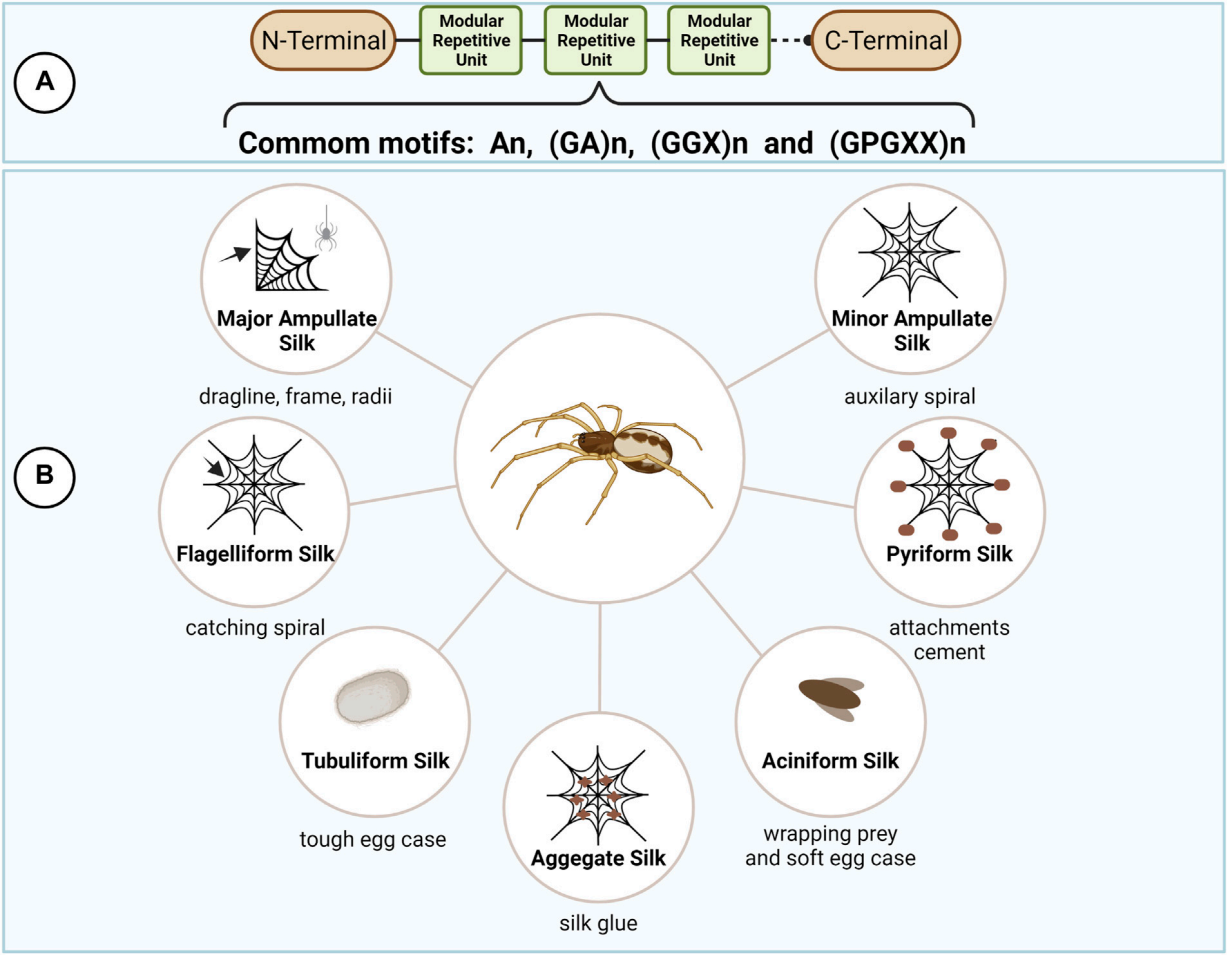
Spider silks types and protein structure. Spider silk proteins (spidroins) are composed by repetitive amino acid motifs flanked by the highly conserved N- and C- terminal domains **(A)**. The different combination of the repetitive motifs in the spidroins directly reflects their mechanical properties, allowing the production of different kinds of silks for specific tasks in spider’s life **(B)**. This figure was created with BioRender.com.

Spider silks’ unique mechanical properties have called the attention of humanity since ancient times (Lewis, 1996). Presenting extreme toughness, through the perfect combination of strength and elasticity, spider silks are formed by a group of proteins commonly named spidroins. They are produced by specialized abdominal glands that are responsible for the assembly of different kinds of silks in charge of specific tasks in spider’s life (Figure 2) (Vollrath, 1992; Lefèvre et al., 2008).

Despite the spider silk type, spidroins are generally 250–350 kDA in size and composed by a highly repetitive core, rich in alanine (A), glycine (G) and proline (P), flanked by the non-repetitive and highly conserved amino (N) and carboxy (C) terminal domains (Xu and Lewis, 1990; Vollrath, 2000; Gatesy et al., 2001; Ayoub et al., 2007; Bittencourt et al., 2007). The modular architecture of spidroins reflects their mechanical properties and is important to fine tune the correct assembly of spider silks in the correct spatiotemporal sequence at the silk gland (Askarieh et al., 2010; Hagn et al., 2010; Tokareva et al., 2014).

The terminal domains are small in size (∼110–130 residues), but both domains fulfill a distinct and essential role in fiber assembly by adopting different conformations in response to changes in pH and ionic concentrations during the spinning process (Knight and Vollrath, 2001). In addition, the terminal domains are crucial for the maintenance of solubility of the spidroins at high concentrations (30–50% w/v) in the silk gland by avoiding the long repetitive regions, largely disordered in solution, to undergo aggregation and premature fiber formation (Hijirida et al., 1996; Hagn et al., 2010; Rising and Johansson, 2015; Andersson et al., 2017; Finnigan et al., 2020). The repetitive core, in contrast, often account for more than 90% of the whole spidroin and are generally composed of short polypeptide motifs which are repeated several times, as follows: poly-A (An), alternating G and A (GA)n, amino acid triplets composed of 2 G and a third variable amino acid (GGX)n, and (GPGXX)n modules, where X represents an variable amino acid residue. These motifs form structural modules such as crystalline β-sheets, β turns, or helices, that underlie the mechanical properties of spider silks (Hayashi et al., 1999; Tokareva et al., 2014; Bittencourt et al., 2021).

Accordingly, orb-weaver spiders can produce up to seven different kinds of silks presenting different combination of polypeptide motifs and, consequently, possessing distinct mechanical properties, including major ampullate silk (MaSp), minor ampullate silk (MiSp), flagelliform silk (Flag), aggregate silk (glue), tubuliform silk (TuSp), aciniform silk (AcSp), and pyriform silk (Hayashi and Lewis, 2001; Hayashi, 2004; Tian and Lewis, 2005; Ayoub et al., 2007; Bittencourt et al., 2007; Perry et al., 2010; Chen et al., 2012; Stellwagen and Burns, 2021). The major ampullate, minor ampullate and flagelliform silks are involved in the production of spider’s orb web. The aggregate glue is responsible for the sticky characteristics of the silk. Female spiders produce the tubuliform silk to make egg sacs. The aciniform silk is used to wrap prey and in the inner part of the egg sac. Finally, the pyriform silk is responsible for the production of the attachment discs that join the web to surfaces.

From these, one of them stands out from the group due to its superior mechanical properties, the MaSp, also known as dragline silk. It is the strongest fiber, with tensile strength compared to Kevlar (one to two GPa) but with higher extensibility (50–60% strain at failure) (Gosline et al., 1999; Vollrath and Knight, 2001; Blackledge et al., 2012). Mostly constituted by two types of spidroins, MaSp1 and MaSp2, it serves as the framework of the spider’s web and it is also used as a lifeline during falls (Xu and Lewis, 1990; Kono et al., 2019). Because of its outstanding mechanical properties, this silk spidroins are the most used for the development of rSSPs.

MaSp1 amino acid composition is characterized by the presence of An, (GA)n and (GGX)n motifs. MaSp2 protein composition is similar to MaSp1, differing only by the presence of the GPGXX motif (Hinman and Lewis, 1992). The An/(GA)n sequence constitutes the hydrophobic crystalline region of the protein, responsible for β-sheet formation, giving dragline silk its high tensile strength. The GPGXX sequence forms the hydrophilic region of the protein and adopts a β-turn conformation. Similar to a spring it provides the excellent elastic properties of the silk. Finally, the (GGX)n motif is the transitional connection between the rigid region and the elastic region of the silk, which will form a 3_10_-helix structure, granting the extraordinary properties of dragline silks (Hayashi et al., 1999; Brooks et al., 2005).

Recently, novel spidroins subtypes were identified as components of dragline silk in some spider species, MaSp3(A/B/C), MaSp4 and MaSp5, the last two were identified only in bark spiders (Garb et al., 2019; Kono et al., 2019; Kono et al., 2021a; Kono et al., 2021b). Focusing in MaSp3-like spidroins, their amino acid sequence is similar to MaSp1 and 2, with high content of G and A, the difference is characterized by the presence of charged amino acid residues, aspartic acid and arginine, in the repetitive region. Interestingly, this spidroin was found to be the most abundant subtype in *Araneus ventricosus*. On the contrary, MaSp4 and MaSp5 have high amounts of P residues present in a unique GPGPQ amino acid motif. Known to confer silk extensibility, the presence of P rich motifs suggests *Caerostris darwini* evolved distinct spidroins capable of increasing its dragline’s toughness, in order to enable the production of its giant orb webs (Garb et al., 2019). According to proteomic data, the abundance of MaSp4 in the *C. darwini* dragline is estimated at 11–16%. Despite their interesting structure, these spidroins were not yet used in any engineering endeavors.

## Recombinant spider silk expression systems

Spiders are territorial and cannibalistic, making farming spiders impractical for their silk. Further, the process of collecting spider silk from live spiders is highly labor-intensive and only results in relatively small quantities of silk per spider being collected. When combined, these obstacles make it nearly impossible to imagine a process of spider farming that is both practical and economical. This has led to a relatively large interest from the research community in the heterologous expression of spider silks in various host species.

**FIGURE 3:**
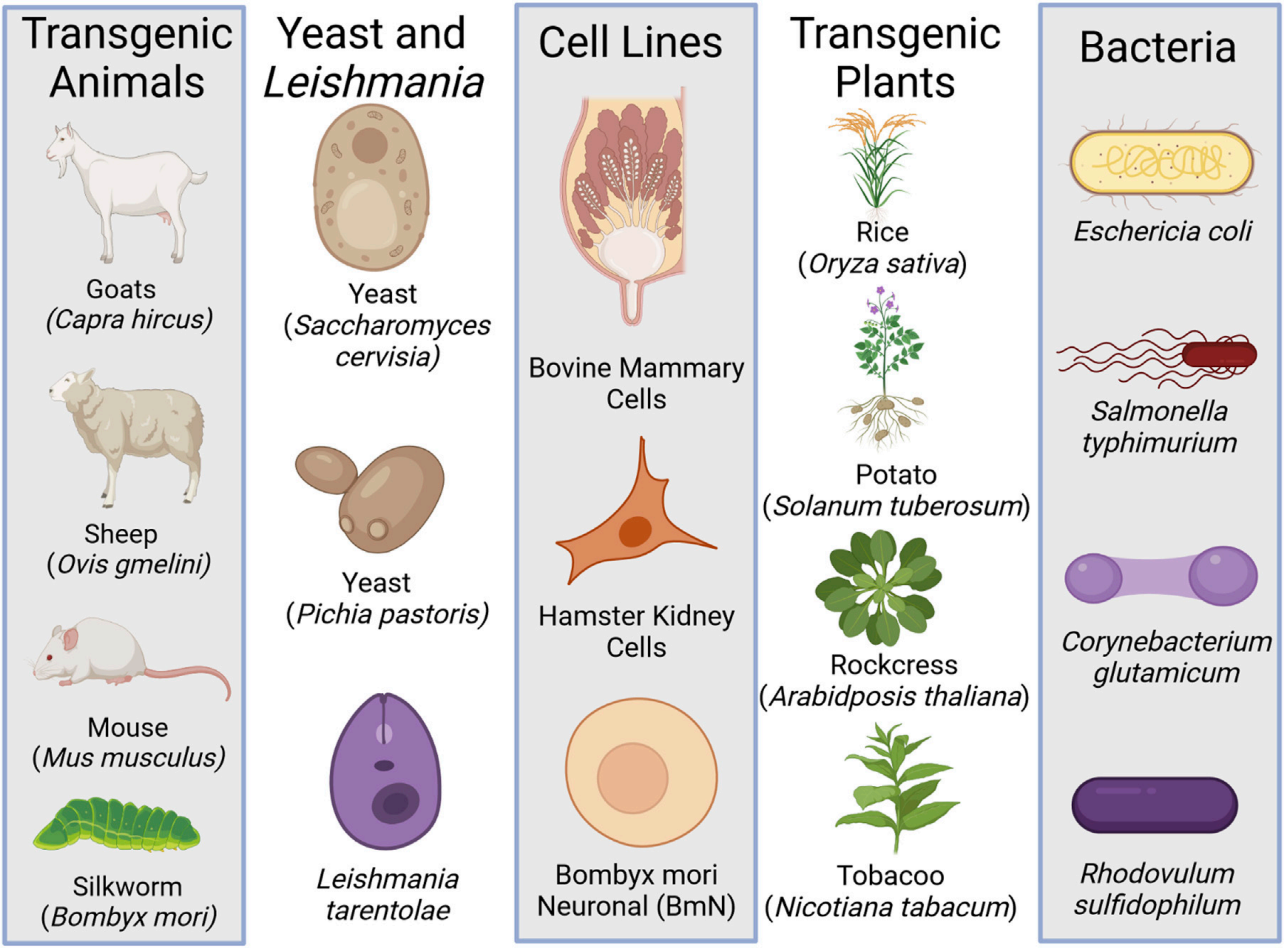
The different heterologous expressions systems used to produce recombinant spider silk protein (rSSP). Created with BioRender.com.

In order to investigate spider silk proteins and their possible applications synthetic biology tools have been applied to genetic engineering a variety of organisms to express the rSSP. Bacteria, yeast, insects, mammals, and plants have all been explored as possible production organisms for rSSP (Figure 3). In all cases, with the exception of silkworms, the rSSP is ultimately produced and purified as a protein powder, not a fiber. Genetically modified silkworms produce spider silk as a minor percentage of their silk fiber (Teulé et al., 2012; Zhang et al., 2019). Expression in hosts that only produce a protein and not a fiber has advantages and disadvantages. The advantage is that organisms such as bacteria, yeast, and plants are generally economical choices for recombinant structural protein expression. Production as protein powder also allows for a myriad of materials to be formed from these proteins that normally form a fiber. Films, adhesives, sponges, foams, and particles have all been reported in the literature (Jones et al., 2015). The disadvantage is primarily that the protein must be purified, which can be costly and time-consuming, and that the rSSP also then must be formulated into a fiber or other material form.

All of the heterologous expression platforms of rSSP have experienced some level of success, but all have failed to reach what is generally considered to be sufficient production levels for engineering applications. This is evidenced by the fact that there are very limited products available, and those products largely do not take advantage of the natural mechanical properties of spider silks.

The challenges of mass production of rSSP must be overcome to realize their remarkable potential. These difficulties arise due to spider silk’s repetitive nature, high molecular weights, and their reliance on two amino acids, glycine, and alanine. This has led to researchers largely producing shortened variants of these proteins, with a couple of notable exceptions (Xia et al., 2010; Bowen et al., 2018).

### Bacteria

Bacteria, particularly *Escherichia coli*, have been some of the most reported and studied expression systems for rSSP. In fact, the first known expression of rSSP was done using *E. coli* in a report by Lewis et al., in 1996. However, generally speaking, low expression levels have plagued the production of rSSP in *E. coli* due to the rSSP’s high molecular weight, repetitive nature, and high reliance on glycine and alanine.

A recent techno-economic analysis of *E. coli* rSSP production using IPTG for induction suggested that 2 g/L of induced bioreactor media was required to overcome the cost of production (Edlund et al., 2018). This is a relatively high benchmark for the production of these proteins due to the outlined problems.

Groups have attempted to overcome these issues by supplementing tRNA to prevent premature translation termination. Xia and colleagues reported an expression system that supplemented tRNAs and could produce 1.2 g’s/L of recovered rSSP (Xia et al., 2010). The recombinant protein expressed was a MaSp1 variant with a molecular weight of 284.9 kDa. Remarkably, they were able to produce near-native molecular weight protein that, when spun into a fiber, had similar mechanical properties to natural spider silk. Interestingly, this work was performed in 2010, 12 years prior to this writing, and this remarkable research has not led to further publications or evidence of translating the system to commercial production.

More recently, a report detailed an expression system that, in some cases, supplemented tRNA pools but did not show marked improvement in rSSP expression levels even when relatively low molecular weight rSSP were produced (Bhattacharyya et al., 2021).

Scheibel and team attempted to express a chimeric rSSP that would result in high yields when expressed in *E. coli* (Thamm and Scheibel, 2017). The authors combined Latrodectus hesperus terminal domains with the Cyrtophora mouccensis core domain. The resulting sequence was reported to be absent in proline, with shorter poly-alanine segments that were not evenly distributed. As a monomer, the chimeric protein was 42.7 kDa. Expression levels were reported to be in the range of 300–400 mg per liter of induced culture. The rSSP was soluble in aqueous buffers from which fibers were spun. Fiber properties were remarkable for a short rSSP.

Producing high molecular weight rSSP is a prominent goal of spider silk research. The publication by Xia et al. provided a report of the possibilities when high molecular weight rSSP are expressed and used to create fibers. However, with the challenges of creating these high molecular weight variants in *E. coli*, alternative methodologies had to be employed. Lin et al. first reported an intein system of protein subunit assembly (Lin et al., 2016). In this study, the authors assembled a MaSp repetitive module with AcSp (aciniform silk protein) module that contained the highly conserved carboxy-terminal domain. These resulting hand-drawn chimeric fibers had impressive mechanical properties and provided the first demonstration that small subunits could be combined into large, more native-length rSSP, using the intein system.

Building on the success of Lin et al., Bowen et al. utilized the split intein system to create 192 repeats of MaSp1 from *Nephila clavipes* (Bowen et al., 2018). The individual subunits were 290 and 282 kDa. The assembled subunits had a molecular weight of 556 kDa and had a yield of 2 g/L or 63 mg/g cell dry weight. Remarkably, when these proteins were spun into fibers, they had similar mechanical properties to natural spider silk. Interestingly, neither the subunit proteins nor the assembled protein contained the native, highly conserved amino and carboxyl termini. Also, in this report, the authors spun the shorter 290 and 282 kDa subunits individually and compared them to the full-length assembled rSSP. The results indicated that the mechanical properties of the full-length assembled rSSP had nearly double the mechanical properties for tensile strength and elastic modulus of the single subunits. Again, this evidence points to the importance of producing rSSP at near-native molecular weights to gain mechanical properties that are similar to natural spider silk.

Other bacteria have also been studied for their ability to produce rSSP. *Corynebacterium glutamicum* was engineered to secrete a MaSp1-like rSSP derived from *Trichonephila clavipes* (Jin et al., 2022). Two proteins were expressed and secreted, a 43 kDa variant and a 168 kDa variant. The 43 kDa MaSp1 was secreted and recovered at 554.7 mg/L, while the 168 kDa variant was secreted and recovered at 68 mg/L. The discrepancy in expression level was likely due to the protein’s repetitive nature and premature termination of translation. The resulting fibers had appreciable mechanical properties but fell short of the mechanical properties of natural spider silk.

*Salmonella typhimurium* and its type III secretion system were also studied for their ability to produce rSSP (Widmaier et al., 2009). The recombinant proteins, derived from the orb-weaving spider *Araneus diadematus*, ranged from 25 to 56 kDa. The authors reported secretion rates of up to 1.8 mg/L/h, with up to 14% of the proteins being secreted.

Expressing rSSP’s has also been attempted in *Rhodovulum sulfidophilum*, a marine photosynthetic purple bacteria (Foong et al., 2020). The authors demonstrated the heterotrophic production of four proteins derived from *N. clavipes* dragline silk, ranging from 7.9 to 20.9 kDa. All proteins were successfully expressed and ranged from 3.4 mg/L to 10.2 mg/L. Comparatively, using *E. coli*, recoveries have been reported up to 1.2 g/L. There are still a number of challenges that need to be resolved in order to use *R. sulfidophilum* as a production platform for rSSP, which are the same or similar challenges faced when using *E. coli* (glycine and alanine, repetitiveness, and molecular weight). However, the use of heterotrophic bacteria may be an essential step in sustainable rSSp production.

### Yeast and *leishmania*

Yeast has also been studied for its ability to produce rSSP. While yeast, like bacteria, is considered a cost-effective and scalable means for production of the rSSP, they suffer from low expression in flask cultures and proteolysis of the target protein (Werten et al., 2019).

Fahnestock and Bedzyk (1997) reported the first demonstration using *Pichia pastoris* to produce spider silk proteins. The authors used an analog of spider dragline silk proteins controlled by the AOX1 promoter. They demonstrated that the rSSP could successfully be produced and then secreted into the extracellular medium.

Jansson and team utilized *Pichia pastoris* to produce an engineered rSSP, Z-4REPCT, that, when expressed and purified, could assemble into silk-like fibers and also bind antibodies using the IgG-binding Z domain (Jansson et al., 2016). However, the purified forms of the protein failed to self-assemble, likely due to aberrant glycosylation of the carboxy-terminal. Additionally, the protein was proteolytically degraded due to a recognition sequence in the carboxy-terminal recognized by proteases from *Pichia pastoris*.

*Saccharomyces cerevisiae* has also been studied for its ability to produce rSSP. Sidoruk et al. (2015). Primarily focused on improving culture conditions to reduce the cost of producing rSSP in yeast. Their efforts were successful, and through process optimization, they were able to reduce the concentrations of sucrose, peptone, and yeast extract. The study did not reveal protein yield, nor did it report purifying the proteins to study material forms and their mechanical properties.

Finally, a report on the expression and secretion of major ampullate spidroin mimics (MaSp1 and MaSp2) in *Leishmania* was reported by Lyda et al. (2017). *Leishmania* was chosen because it offers several advantages over other systems, such as rapid growth, high optical densities, and simple media formulations. The study successfully demonstrated secretion of the rSSP that was purified and spun into fibers. However, the authors say that a major concern of using *Leishmania* is low rSSP yield.

### Insect and mammalian cell lines

Mammalian cell lines have seen limited exploration for the production of rSSP. The proteins MaSp1 and MaSp2 derived from *N. clavipes* and ADF-3 from *A. diadematus* were expressed in both bovine mammary epithelial cells and hamster kidney cells (Lazaris et al., 2002). Both cell lines secreted soluble forms of the proteins (60–140 kDa) that could be purified. From ADF-3 protein (60 kDa), fibers were spun that had mechanical properties that was described as approaching that of natural spider silk. However, the fibers tested only had a tenacity that was 1/3 to 1/5th that of the control natural spider silk.

A later study also sought to express rSSP, a major ampullate spidroin one gene fragment from *Euprosthenops sp*, in the mammalian cell line derived from the African green monkey kidney, COS-1 (Grip et al., 2006). While successful, the authors reported very low expression of the rSSP and did not report protein purification that could result in fiber or other material formation.

Insect cells have also been explored as production platforms for rSSP. *Bombyx mori* neuronal (BmN) cells have been explored for their ability to produce these proteins (Zhang et al., 2008). The BmN cell line incorporated a 70 kDa MaSp1 fused with eGFP. The fusion protein was observed in the BmN cells and occupied approximately five percent of the total protein of the cell.

Tissue culture is inherently expensive. For therapeutic proteins, this can be overcome due to the value of the produced proteins. However, for structural proteins, it does not seem possible or reasonable that cell culture will be a viable method of production due to the very high quantity of protein required for even small engineering applications.

### Transgenic plants

Plants are another heterologous expression system that is both scalable and economical. Utilizing plants has advantages in genetic stability and the ability to produce large proteins without expensive machinery requirements used for insect, mammalian, bacterial and yeast production. However, expression of recombinant proteins in plants does not result in high protein yields (Schillberg et al., 2019). Nonetheless, a variety of plants have been studied for their ability to produce rSSP: Tobacco, potatoes, rockcress (*Arabidopsis*), and alfalfa.

In 2001, Scheller et al. reported the first production of rSSP in plants (Scheller et al., 2001). The study incorporated synthetic spider silk genes from 420–3,600 base pairs in both tobacco (*Nicotiana tabacum*) and potatoes (*Solanum tuberosum*). The rSSP represented up to 2% of the total soluble protein in both plant species. The proteins were targeted to the endoplasmic reticulum in the leaves of tobacco and potato and also to the potato tuber, but the rSSP was only successfully expressed in the leaves of tobacco and the potato tuber. The authors reported that rSSP up to 100 kDa could be detected in these plant tissues.

Menassa et al. built upon the Scheller tobacco rSSP expression study results and expressed a MaSp1 of 60 kDa and a MaSp2 of 58 kDa, which were derived from *N. clavipes* (Menassa et al., 2004). They demonstrated that both of the proteins could successfully be produced in the leaves of the transgenic tobacco plants. They also took their experimentation a step further and conducted a field trial that demonstrated the rSSP’s could be produced at an agricultural scale. The authors stated that protein expression was low at 0.01% of the total soluble protein, which is much lower than the Scheller study at 2% of the total soluble protein.

More recently, in 2016, two studies used tobacco to express rSSP. The first by Weichert and team sought to express recombinant flagelliform (FLAG) in the seeds of the tobacco plant (Weichert et al., 2016). The study expressed small variants of FLAG that assembled into multimers using the intein system. Multimers larger than 460 kDa were produced from a 37.6 kDa monomer. The maximal accumulation of the FLAG multimers was 190 ug/g fresh weight of seeds. Uniquely, the multimer rSSP was stable in the seed, suggesting that the seeds could be stored for up to 1 year.

The second study that sought to express rSSP in tobacco was performed by Peng et al. (2016) also utilized the intein system to create recombinant MaSp1 and Masp2 variants that included the amino and carboxy-terminal domains, and they ranged in molecular weight from 73 to 162 kDa. This study, however, targeted the rSSP to the leaves of the tobacco plant. The yield ranged from 0.74 to 1.89 percent of the total soluble protein for smaller multimers (73 and 81 kDa).

Alfalfa (*Medicago sativa*) is a common crop and an ideal host system to produce recombinant proteins due to its availability and also that it can be harvested multiple times in a single year. An attempt to express recombinant MaSp2, ranging in size from 80 to 110 kDa in the leaves of alfalfa, was performed by Hugie (2019). Unfortunately, the protein was unrecoverable from the leaves, likely due to processing (freezing). However, the proteins were expressed, indicating some potential in utilizing high yield crops such as alfalfa for rSSP production.

*Arabidopsis* (rockcress) has also been studied for its ability to express rSSP. *Arabidopsis* are small flowering plants and was the first plant to have its entire genome sequenced, making it an intriguing organism for heterologous expression of rSSP. In 2004, Bar et al. expressed a 64 and 127 kDa silk-like protein inspired by MaSp1 from *N. clavipes* (Barr et al., 2004). The gene sequences were introduced into *Arabidopsis* for both leaf and seed-specific expression. The results indicate that expression in the leaves and seeds was successful. Seeds produced 1.2% of the total soluble protein of the 64 kDa protein and 0.78% of the total soluble protein of the 127 kDa. Leaves produced 0.34% of the 64 kDa protein and 0.03% of the 127 kDa protein. The authors suggest that seed production in Arabidopsis is more desirable due to the higher rSSP accumulation levels, seed storage, and processing conditions.

In 2005, Yang and colleagues expressed two synthetic analogs of spider dragline silk protein of 64 kDa in molecular weight in *Arabidopsis* (Yang et al., 2005). When targeted to the apoplast and endoplasmic reticulum, yields were observed to be as high as 8.5% of the total soluble protein in leaf tissue. When targeted to the ER lumen and the vacuole, yields were observed as high as 18% of the total soluble protein of the seeds.

### Transgenic animals

There have been several reports of using silkworms, goats, and sheep to express rSSP’s. Silkworms have an obvious advantage in that they are the only heterologous expression platform that is capable of spinning fiber. In addition, an industry has been in place for thousands of years to rear silkworms, harvest the cocoons, and process the silk fibers. For fibers, this makes them an ideal choice of rSSP production. Goats, on the other hand, cannot spin a fiber, but they can produce relatively large volumes of rSSP laden milk. Sheep are a relatively new entry into transgenic animals expressing spider silk which not nearly as advanced as silkworms or goats.

The first report of genetically modified *B. mori* came in Wen et al. (2010). The authors piggyBac system was utilized and a 1.5 kb MaSp1 gene sequence was incorporated, driven by the sericin 1 promoter. The piggyBac system was delivered via microinjection of the eggs, a process that is inefficient and time-consuming. However, the authors were successful in their effort, and transgenic silkworms were confirmed. Successful incorporation of the MaSp1 protein in the silkworm fiber was confirmed via western blot. The resulting transgenic silk fiber had improved mechanical properties. The ultimate stress increased from ≈575 MPa to ≈675 MPa and the strain improved from ≈15% to nearly 20%. While improved mechanical properties were observed, the transgenic silkworm fibers were still far below the mechanical properties of natural spider silk.

A second report, in 2012, utilized the piggyBac system to incorporate spider silk genes in *B. mori*, which utilized a synthetic spider silk sequence not found in nature (Teulé et al., 2012). The rSSP sequence contained both elastic and strength motifs and was 2,463 bp, the GFP gene was added for easy identification of positive cocoons. The glands and cocoons of the positive transgenic silkworms were very clearly green fluorescent. And the fiber mechanical properties were higher than observed in the previous study with the very best specimens having a toughness equivalent to that of natural spider silk. Refinements of genetic modification techniques led to transgenic silkworms expressing rSSP using the transcription activator-like effector nuclease (TALENs) system. Using the TALEN system, the individual gene sequence for the proteins in silk fibroin could be targeted. In 2018, Xu et al. utilized TALEN-mediated insertion of a 67 kDa MaSp1 analog derived from *N. clavipes* (Xu et al., 2018). The authors were able to replace the silkworm fibroin heavy chain gene with their synthetic MaSp1 gene via homology-directed repair. The study reported that up to 35.2% of the resulting cocoon shell consisted of the synthetic MaSp1 protein. However, the mechanical properties of the transgenic fibers were mixed. In comparison to the wild-type control, the breaking stress, and Young’s modulus were decreased while the strain and energy to break were increased. The authors speculate that the mixed mechanical results could be due to the incorporation of a relatively small rSSP.

CRISPR/Cas has also been used to generate silkworms that express spider silk as a portion of their silk fiber. In 2019, Zhang et al. reported incorporating native-sized rSSP into an intron of either the light or heavy chain of *B. mori*. Both MaSp1 and MiSp1 were utilized coupled to eGFP to identify positive transformants at the cocoon stage. Multiple generations of positive transgenic silkworms were created, indicating stable gene integration. The best fiber mechanical properties were observed from third-generation silkworms, MiSp1 in the heavy chain, which had a tensile strength that exceeded natural dragline silk from *N. clavipes*. Enough transgenic silk fibers were collected from their experiments that they were able to produce a pair of knitted gloves.

Mammalian systems have also been explored to produce rSSP as a purified protein powder. Initially, and as a means to validate the mammalian expression of rSSP in milk, mice were utilized (Xu et al., 2007). Xu et al. demonstrated that not only could mice be created but that the rSSP could be targeted to the milk via casein promoter. While the dragline-like proteins were not purified, they were readily observable in the milk through western blot procedures. The maximum rSSP level measured in the milk was 11.7 mg/L.

Goats (*Caprus hircus*) have also been used to express recombinant spider silk proteins, again when targeted to the mammaries and the milk. Although there are no publications that detail their generation, it has been reported that the goats could produce as much as 0.5 g/L or milk and that the purification process utilized tangential flow filtration (Tucker et al., 2014; Yazzie et al., 2017).

The first publication that detailed the purification process from transgenic goats appeared in 2015 by Tucker et al. (2014). Thin films were created and processed from the rSSp purified from the goat milk. A second publication in 2015 also utilized goat-derived rSSP, which demonstrated a process for spinning those proteins into fibers with appreciable mechanical properties (Copeland et al., 2015). The third publication in 2015 demonstrated a method by which to solvate the goat-derived rSSP using heat and pressure from which multiple material forms could be obtained (Jones et al., 2015). Finally, in 2016 Harris et al. utilized rSSP from goats to produce a breadth of material forms that were mechanically and physically characterized (Harris et al., 2016).

Recently, there has been a report of incorporating rSSP into sheep embryos which was targeted for expression in the hair follicle (Li et al., 2021). Unfortunately, none of the implanted embryos resulted in live fetuses. So, rSSP expression levels could not be observed.

## Bioengineering of spider silks for biomedical applications

The relationship between the modular sequence architecture of spidroins and the mechanical properties of silks have allowed the production of rSSP in different host systems for a myriad of applications. Besides that, once produced, rSSP can be assembled in different structures such as fiber, film, foam, non-woven mashes and spheres (Teulé et al., 2007; Spiess et al., 2010; Jones et al., 2015).

**FIGURE 4:**
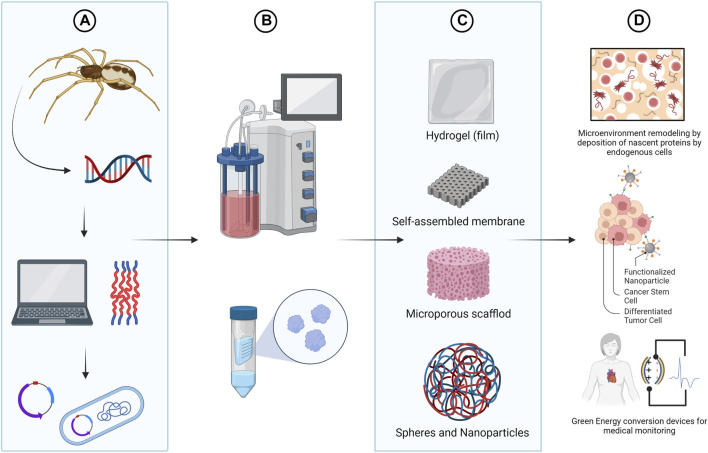
Bioengineering of spider silks. Using synthetic biology tools it is possible to design spider silks with tailor made functionalities **(A)** and to produce recombinant spider silk protein (rSSP) using heterologous expression systems **(B)**. Although in nature only fibers are formed from spidroins, *in vitro*, rSSP can be molded in different morphologies including nanofibrils, particles, capsules, hydrogels, films or foams **(C)**. The versatility of spidroins, along with their biocompatible and biodegradable nature, also place them as the perfect biomaterial for the design of improved drug delivery systems, tissue engineering scaffolds and biosensor devises **(D)**. Created with BioRender.com.

The versatility of spidroins, along with their biocompatible and biodegradable nature (Dams-Kozlowska et al., 2013; Müller-Herrmann and Scheibel, 2015; Deptuch et al., 2021), expand silk technologies to develop innovative bio-derived structural materials and place them as leading-edge biological macromolecules for various biomedical applications (Altman et al., 2003; Long et al., 2021). Furthermore, through synthetic biology, it is possible to bioengineer rSSP with specific functional motifs, for the development of improved drug delivery systems, scaffold for tissue engineering and wound dressing (Schacht and Scheibel, 2014; Nguyen et al., 2019; Salehi et al., 2020; Liu et al., 2021; Zhang et al., 2021). More recently, rSSP are also being explored for use in a wide range of novel green energy conversion devices for potential biomedical applications, such as the development of implantable organ monitors (Zhang et al., 2018; Huang et al., 2020) (Figure 4).

### Tissue engineering and regenerative medicine

Tissue engineering is a field of medicine that uses interdisciplinary methods to formulate biological systems to aid in the growth of new cells and tissues in order to maintain and improve tissue functions, as well as to mimic whole organs to model diseases and screen for therapeutic drugs (Lynch et al., 2021).

The biggest challenge in tissue bioengineering is to recreate the extracellular matrix (ECM), the natural environment in which the cells normally grow (Yue, 2014; Lynch et al., 2021). In this matter, the utilization of biopolymers, as spider silks, has become the favorable option as they are biocompatible, biodegradable and do not generate an immune response when placed into an organism (Asghari et al., 2017; Zhang et al., 2021). However, for scaffold design many factors must be carefully considered to mimic the function and tissue specific three-dimensional network of the ECM in order to provide a favorable microenvironment for functional tissue regeneration (Bakhshandeh et al., 2017).

For instance, silk proteins generally lack bioactive peptides for enhancing cell functions. To further improve interactions between cells and spider silk based materials, the cell binding peptide motif RGD (Arg - Gly - Asp) from fibronectin was genetically incorporated in rSSP and spun into films to support osteoblastic differentiation (Morgan et al., 2008). The authors observed that higher amounts of β-sheet content improved film stability and, consequently, osteoblast attachment and differentiation.

In another study, the engineered spider silk protein eADF4(C16) (based on the spidroin ADF4 spidroin from the spider *A. diadematus*) was modified with the integrin recognition sequence RGD by a genetic (fusing the amino acid sequence GRGDSPG) as well as a chemical approach (using the cyclic peptide c (RGDfK)) (Wohlrab et al., 2012). After film processing it could be demonstrated that adhesion and proliferation of fibroblasts were significantly improved on films made with both RGD-modified proteins. The same result was observed for human induced pluripotent stem cell (hiPSC)-derived cardiomyocytes (Esser et al., 2021). While hiPSC-cardiomyocytes could not adhere to eADF4(C16) without additional modification, they could be cultivated on specifically engineered spider silk variants, including eADF4(C16)-RGD. Interestingly, the hiPSC-cardiomyocytes also exhibited selective cell behavior on films made with different rSSP.

Another rSSP, encoding four repetitive poly-Ala and Gly rich blocks followed by the C-terminal domain of *Euprosthenops australis* MaSp1 (4RepCT), was genetically functionalized with different cell binding peptides (RGD, IKVAV and YIGSR) and casted as films to form synthetic EMC (Widhe et al., 2013). Four different human primary cell types; fibroblasts, keratinocytes, endothelial cells and Schwann cells, were applied to the matrices formed by rSSP. Similarly to the hiPSC-cardiomyocytes, the cellular adherence of each cell investigated under serum-free culture conditions varied between matrices. Showing that the cell sensibility in response to specific cell binding peptides must be considered (Johansson and Rising, 2014).

Leal-Egaña et al. (2012) also investigated the interaction between fibroblasts (BALB/3T3) and surface topology of different scaffolds made of the recombinant spider silk protein eADF4(C16). The BALB/3T3 cells on hydrogels and films made of eADF4(C16) showed low cell adhesion without observable duplication. Electrospun non-woven scaffolds however, enabled both the adhesion and proliferation of the cells. Since eADF4(C16) lacks specific motifs for cell attachment, these results indicated that the topography of a silk scaffold also influences cell-ECM interaction responses.

The feasibility of rSSP attached to RGD-tags was likewise examined as a wound-dressing material for coverage of deep second-degree burn in animal models (Baoyong et al., 2010). The results of implantation testing showed that wound healing in the treatment groups was much better than that in the control. The rSSP scaffolds promoted the recovery of wound skin by increasing the expression and secretion of basic fibroblast growth factor and synthesis of hydroxyproline. Additionally, non-woven nanofiber matrices were also pointed as the best scaffold to enhance skin regeneration (Min et al., 2004; Li et al., 2015).

More recently, Pawar et al., 2019 evaluated rSSP based nonwoven mesh applied in the field of nerve repair. In this research, the surface structure of spidroin mesh provided the ideal attaching sites for nerve regeneration too, showing no cell toxicity. These results together suggest that nonwovens mesh might be the best morphology for the production of spider silk based scaffolds for tissue engineering. Probably due to the higher availability of cell attachment surface in its microporous surface structure, allowing a faster cell adhesion, proliferation and the transportation of molecules around the fibers.

Nevertheless, recent studies have qualified rSSPs nanomembranes as synthetic basement membrane, a pliable sheet-like type of ECM (Kruegel and Miosge, 2010). Harris et al. (2019) have shown the capacity of rSSPs, based on MaSp1 and MaSp2 sequences, to function as substrate for a synthetic Bruch’s membrane and model the retinal pigment epithelium. Comparisons to native structures revealed that the rSSP membranes exhibited equivalent thicknesses, biomechanical properties and barrier functions.

Another study used the 4RepCT silk protein functionalized with the RGD-containing cell binding motif to produce 470 ± 110 nm thin nanofibrillar membranes. The scaffold supported a cell co-culture into an *in vitro* blood vessel wall model (Tasiopoulos et al., 2021). The rSSP nanofibrillar membranes allowed the formation of a confluent endothelium on the apical side of the membrane, with the ability to regulate the permeation of representative molecules of dextran (3 and 10 kDa) and IgG. A thicker ECM was also formed on the basolateral side by the smooth muscle cells, with enhanced barrier properties compared to conventional tissue culture inserts. This result points to possible use of genetically functionalized rSSPs to develop organ-on-a-chip systems (Fritschen and Blaeser, 2021).

### Drug delivery systems

It is known that the use of nanostructured systems for controlled drug delivery has several advantages over traditional administration, such as: 1) better pharmacokinetics of the drug; 2) decreased toxicity to healthy organs; 3) possible facilitation of preferential accumulation and uptake by target cells; and 4) programmed drug delivery profile (Zhang et al., 2013; Youssef et al., 2019). In addition, the use of biomaterial carriers in drug delivery has the ability to reduce the amount of drug administered, improving drug bioavailability and therapeutic responses.

Several biologically-derived polymers, including spider silks, have already been used to produce therapeutics and vaccine delivery systems (Numata and Kaplan, 2010; Shi et al., 2016; Jao et al., 2017; Crivelli et al., 2018; Hong et al., 2018; Idrees et al., 2020). One of the greatest advantages of spider silk is its hydrophobicity. Hydrophilic biomaterials in aqueous solutions can quickly dissolve and therapeutic burst release may occur (Adeosun et al., 2020). Besides that, the ability of these proteins to self-assemble into higher-order structures allows for the development of hybrid nanoparticles with custom-designed properties encoded genetically.

Different studies have proven the ability of nanoparticles formed from rSSP to function as carriers for the release of different active principles (Blüm and Scheibel, 2012; Doblhofer and Scheibel, 2015; Kucharczyk et al., 2018). Lammel et al. (2011) demonstrated that nanoparticles developed from the heterologous eDF4(C16) spidroin were stable and could efficiently bind and release low molecular weight model drugs. eADF4(C16) particles have also been successfully evaluated as carriers of high molecular weight drugs. Drugs such as doxorubicin (anthracycline family antibiotic) could also be efficiently administered and released into cells when spider silk particles were used as a vehicle (Schierling et al., 2016).

Other studies have also demonstrated that, through genetic engineering, it is possible to alter the amino acid sequence of spidroins to control their properties and, consequently, their affinity for drugs (Szela et al., 2000; Kucharczyk et al., 2018). For instance, Doblhofer and Scheibel (2015) replaced a glutamic acid residue with lysine (L) in the amino acid sequence of the protein eADF4(C16). The newly derived silk protein, eADF4 (κ16), was positively charged, opposite to the original eADF4(C16). This modification allowed the encapsulation of negatively charged active principles, such as DNA particles. The modification of silk protein by adding the poly-L nucleic acid binding domain also enabled the development of novel oligonucleotide delivery systems (Numata et al., 2010; Kozlowska et al., 2017). These strategies can be utilized to improve pharmacokinetics of DNA/RNA-based therapeutics applied for cancer immunotherapy and nucleic acid vaccines.

Furthermore, functionalized bioengineered rSSPs incorporated with cell penetrating peptides and/or receptor interacting motifs had the ability to enhance cellular uptake and target specificity of the delivery systems in cancer cells (Numata and Kaplan 2010; Numata et al., 2011; Florczak et al., 2014; Elsner et al., 2015). A ErbB tyrosine kinase receptor family functionalized rSSP spheres have also shown efficiency for cancer treatment *in vivo* (Florczak et al., 2020). The same strategy was also used to add a desired peptide sequence in the rSSP for the development of peptide vaccines, avoiding premature degradations of the target peptide and proper cytotoxic T-cell activation (Lucke et al., 2018).

In a more recent study, the functionalization of rSSPs was acquired by a chemical approach (Mulinti et al., 2021). The MaSp1 based rSSP nanosphere was chemically embedded with a thrombin-sensitive linker and was able to release the antibiotic vancomycin only at the site of infection, allowing the development of a “smart” delivery system. This strategy may mitigate multidrug resistance by bacteria and preserve the lifetime of our current antibacterial agents.

Although spheres and nanocapsules are the morphologies of choice for drug delivery, initial studies using the eADF4(C16) spidroin also indicated the potential use of injectable rSSP hydrogels for the delivery of therapeutic molecules (Kumari et al., 2018). Additionally, *in vitro* release testing showed a correlation between protein release and electrostatic interactions with the hydrogel, pointing to a possible application in localized therapies and sustained release applications.

### Biofunctional green energy conversion devices

The versatility and multifunctionality of spider silk goes further than just mixing and matching the different properties of silk desired for a specific application, it can be placed as a key polymer in bridging the gap between sustainable materials and sustainable energy conversion for biomedical applications (Strassburg et al., 2021).

For instance, devices for physiological signal monitoring such as in electrocardiography, electroencephalography, and to collect evoked potentials data, are made of materials which are expected to be soft, hydrophilic, and electroconductive and are designed to minimize the stress imposed on living tissue during monitoring (Tsukada et al., 2012)

Spider silks is an ideal candidate to fulfill these requirements. Besides their excellent mechanical properties and biocompatibility, spider silks are capable to produce piezoelectricity, which is the ability of a material to generate an internal electric field when subjected to mechanical stress or strain (Berlincourt, 1971). A recent work from Karan et al. (2018) quantified the structure-dependent piezoelectric response of silk from the spider species *Nephila pilipes* as up to ≈0.36 p.m./V and produced an effective bio-piezoelectric nanogenerator using the native silk.

Nevertheless, spider silks can be genetically designed and processed with specific and enhanced properties, including the changing of the residual electric charge and the introduction of artificial binding sites on the proteins, to permit improved optical and electronic properties with extra biological functionalities on demand (Currie et al., 2011; Herold et al., 2019). This opens up the possibility for novel energy conversion devices that are easy to manufacture, environmental friendly and durable to be applied *in vivo* conditions.

Spider silks are also suitable for constructing triboelectric nanogenerators (TENG). TENGs employ the combination of triboelectric effect, which is the process of electron transfer and re-equilibrium between two friction layers, and electrostatic induction to convert mechanical energy into electrical energy (Wang, 2013). Zhang et al. (2018) demonstrated that larger rSSPs presented enhanced triboelectric output and developed a biologically functional TENG with well-define mechanical and chemical properties. They designed rSSP-based TENG to serve as drug-free “antibacterial patch”. The nanocomposites presented not only the antibacterial performance but also biocompatibility in rats. As a proof-of-principle, they also mix the rSSP patch with graphene (GR) and carbon nanotube (CNT) to modulate the bulk property, as well as add Au and Ag nanoparticles for surface modifications. Compared with pure GR, CNT, Au and Ag paired with poly (ethyleneterephthalate), rSSP leveled up the output performance by donating electrons. Moreover, the presence of GR and Ag nanoparticles enhanced the rSSP patch antibacterial properties too.

More recently, a modified concept, known as tribo-piezoelectric nanogenerators (TPNGs), which uses tribo and piezoelectric components to enhance the energy conversion efficiency, was applied to fabricate hybrid devices using rSSPs and poly (ethylene terephthalate)/poly (vinylidene fluoride) (PVDF) (Huang et al., 2020). More importantly, since the rSSPs and PVDF are both biocompatible, a small-scale sRRP/PVDF-GR based TPNG was fabricated (1.5 × 1.5 cm) to monitor and harvest energy from the heart beats of rats. With the beating of the heart, the TPNG was compressed and released, thus generating current flow. Accordingly, it is possible to envision that the rSSP TPNGs could be applied for the development of a number of implantable devices to monitor the movements of various human tissues for therapeutic and diagnostic applications.

## Concluding remarks

The relationship between molecular composition, secondary structures and mechanical properties of spider silks opens the possibility to fully control the molecular characteristic of rSSPs and to develop specific modified spider silk variants.

On the top of that, spider silks have shown great biocompatibility and low immunogenicity *in vivo*, making them attractive candidates for different biomedical applications. For instance, the appropriate design of the spider silk-based biomaterials, tailored to specific therapeutic requirements, provides a favorable environment for the incorporation of this biomaterial into tissue engineering and drug delivery systems. Furthermore, the unique optical and electronic properties of spider silks can also favor the development of biofunctional green energy conversion devices with considerable potential for *in vivo* applications in self-powered medical monitoring and treatment.

Advances in synthetic biology tools have immensely benefited the bioengineering and production of rSSP for biomedical applications. However, further improvements on the production systems are still necessary to overcome the challenges to bring down the costs and achieve cost-efficient large-scale production.
